# Tunable Ordered Nanostructured Phases by Co-assembly of Amphiphilic Polyoxometalates and Pluronic Block Copolymers

**DOI:** 10.1021/acs.nanolett.2c03068

**Published:** 2023-02-16

**Authors:** Andi Di, Jipeng Xu, Thomas Zinn, Michael Sztucki, Wentao Deng, Anumol Ashok, Cheng Lian, Lennart Bergström

**Affiliations:** † Department of Materials and Environmental Chemistry, Stockholm University, Stockholm 106 91, Sweden; ‡ School of Chemistry and Molecular Engineering, East China University of Science and Technology, Shanghai 200237, China; § ESRF, The European Synchrotron, 71 Avenue des Martyrs, CS40220,38043 Grenoble Cedex 9, France; ∥ College of Chemistry and Chemical Engineering, Central South University, Changsha 410083, China

**Keywords:** Amphiphilic polyoxometalate, Co-assembly, Ordered nanostructure, Catalysis

## Abstract

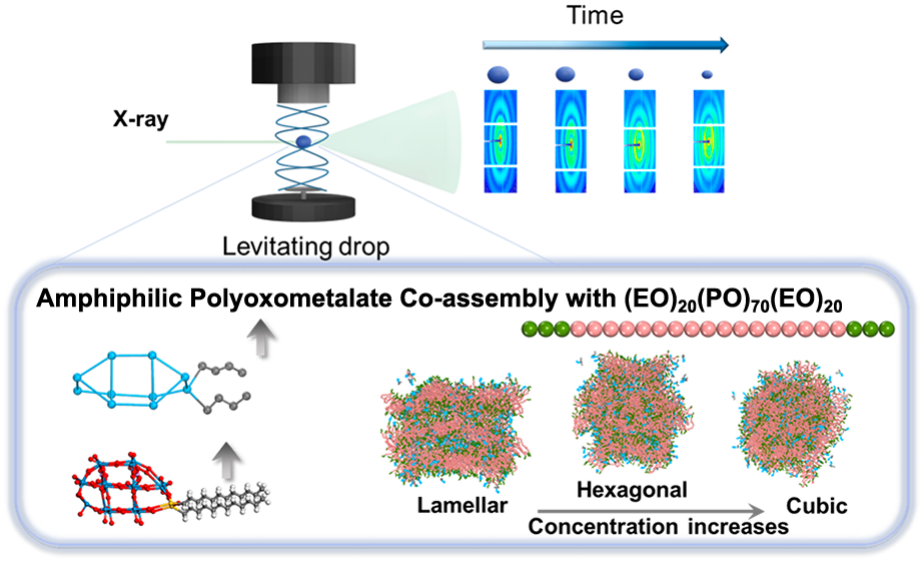

The assembly of polyoxometalate (POM) metal–oxygen clusters into ordered nanostructures is attracting a growing interest for catalytic and sensing applications. However, assembly of ordered nanostructured POMs from solution can be impaired by aggregation, and the structural diversity is poorly understood. Here, we present a time-resolved small-angle X-ray scattering (SAXS) study of the co-assembly in aqueous solutions of amphiphilic organo-functionalized Wells-Dawson-type POMs with a Pluronic block copolymer over a wide concentration range in levitating droplets. SAXS analysis revealed the formation and subsequent transformation with increasing concentration of large vesicles, a lamellar phase, a mixture of two cubic phases that evolved into one dominating cubic phase, and eventually a hexagonal phase formed at concentrations above 110 mM. The structural versatility of co-assembled amphiphilic POMs and Pluronic block copolymers was supported by dissipative particle dynamics simulations and cryo-TEM.

Polyoxometalates (POMs) are polynuclear metal-oxo clusters that can host a range of noble metal atoms to provide a well-defined coordination environment of single-atom sites.^1−3^ The potential applications of single-atom catalysis of POMs have attracted significant research interest due to their versatility and potential for very high atomic utilization and minimal use of scarce and/or potentially toxic metals.^4−6^ It is important to avoid agglomeration and the formation of dense and disordered structures that limit the accessibility of the single-atomic catalytic sites, but the preparation of ordered nanostructures with single-atom sites remains a challenge.^7,8^

Assembly from solution is a powerful and extensively used approach to prepare periodic nanostructures from oligomeric,^9^ polymeric,^10^ and nanoparticle^11−13^ building blocks. Surfactant-encapsulated POMs (SEPOMs) have resulted in the formation of ordered structures in organic solvents^8,14−16^ and have also facilitated recovery.^17,18^ However, the stability of the physically bonded SEPOMs is poor, and the assembly of SEPOMs approach is limited to low concentrations and low POM content.^19^

Grafting organic molecules to form covalently linked POM-organic hybrids avoids some of the issues related to the physically encapsulated SEPOMs and may improve compatibility and processability. Covalently linked POM-organic hybrids have been used to prepare periodic POM-containing nanostructures both in solid state^20,21^ and in organic solvents.^19,22,23^ However, studies of POM-containing nanostructures in aqueous solutions, which offer high single-atom accessibility compared to the reversed micelles in organic solvents, are sparse.^22,24^

Here, we present a study on how periodic nanostructures containing single-dispersed organo-functionalized Wells–Dawson polyoxometalates can be prepared by co-assembly with a Pluronic block copolymer (P123) in aqueous solutions. Covalent grafting of two hydrophobic hydrocarbon chains resulted in amphiphilic Wells–Dawson polyoxometalates (K_6_[P_2_W_17_OSi_2_(C_12_H_25_)_2_], denoted as POM-2C_12_) that could be dissolved in water without agglomeration or precipitation. The phase behavior and formation of ordered nanostructures by co-assembly of P123 and POM-2C_12_ in aqueous solutions were investigated by time-resolved small-angle X-ray scattering (SAXS) and wide-angle X-ray scattering (WAXS) in levitating drops in combination with dissipative particle dynamics simulations and cryo-transmission electron microscopy (cryo-TEM). The use of shrinking levitating drops allowed a wide concentration range to be probed, revealing that the co-assembly strategy resulted in the formation of large vesicles, a lamellar phase, a cubic phase, and eventually a hexagonal phase with increasing concentration. The rich structural diversity and the demonstration of tunable manipulation of ordered POM-based nanostructures by co-assembly could serve as a basis for the production, possibly by supercritical drying,^25^ of structured POM-based materials.

Time-resolved SAXS/WAXS measurements (refer to Figure 1a for the experimental setup) of the aqueous solutions in the levitating droplet (Figure 1b) was used to follow in situ the evolution of the nanostructures in the shrinking aqueous droplet that contains POM-2C_12_ and P123. The molecular structure of POM-2C_12_ is illustrated in Figure S1; the molecular structure was confirmed by C–H correlated NMR; see Figure S2. The molar concentrations in the droplet were calculated according to the volume of the shrinking droplet, and we followed the co-assembly behavior up to the concentration of 115.0 mM (Figure 1c). The minor fluctuations of the concentration and volume are mainly related to the acoustic-field-induced fluctuations of the droplet shape.

**Figure 1:**
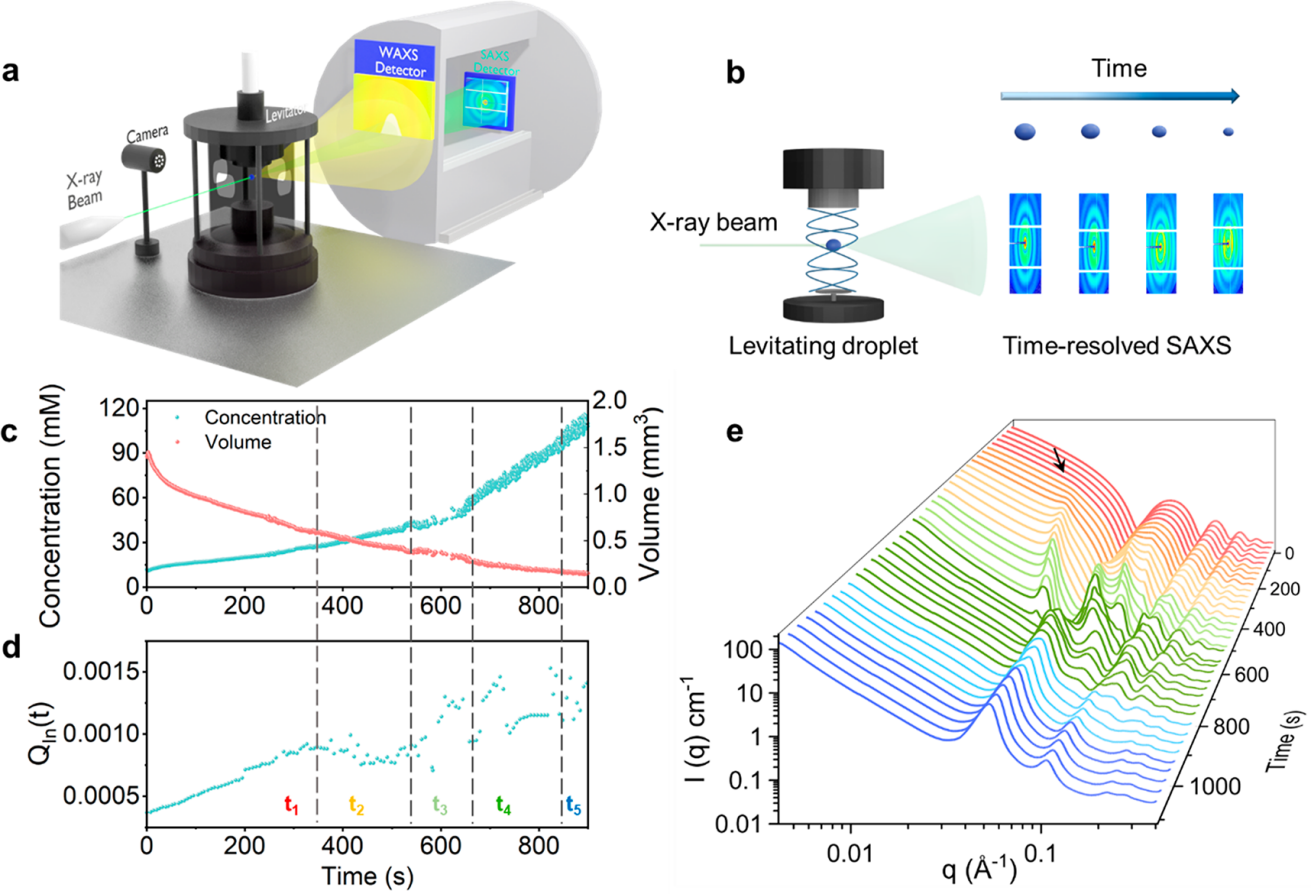
Time-resolved SAXS/WAXS measurements of levitating POM-2C_12_/P123 aqueous drops. (a) Experimental setup for the acquisition of time-resolved SAXS/WAXS and droplet size data. (b) Levitating drop and time-resolved SAXS patterns and images of the levitating drop. (c) Volume and concentration of the shrinking levitating drop with time. (d) Partial scattering invariant (*Q*_*ln*_*(t)*) as a function of time (*t*) and identification of five time regions: ***t*_*1*_** to ***t*_*5*_** for the POM-2C_12_/P123 system with the mixing ratio 1:3. (e) Time-resolved SAXS patterns. Patterns are colored according to the phase of the nanostructures: the red, orange, light green, dark green, and blue regions correspond to ***t*_*1*_**, ***t*_*2*_**, ***t*_*3*_**, ***t*_*4*_**, and ***t*_*5*_** in Figure 1d, respectively. The arrow indicates the time when a correlation peak starts to appear during the measurement.

The partial scattering invariant (*Q*_*In*_*(t)*) is a measure of the total scattering power of the sample^26−28^ and was calculated through the expression *Q*_*In*_*(t) = ∫I(q)q*^2^*dq* (*q* ranges between 0.00416 and 0.4472 Å^–1^) of the 1D reduced data as detailed in the calculation section in Supporting Information (SI). *Q*_*In*_*(t)* is a constant directly proportional to the mean-square average fluctuation in the scattering length density (*Δρ*) and the phase composition (*ϕ(*1 *– ϕ)*, *ϕ* is the volume fraction of the nanostructures in the solution). The *Δρ* was kept constant for a specific solution system; thus, the apparent variation of *Q*_*In*_*(t)* can be used to probe the phase boundaries.^29^ The time-resolved *Q*_*In*_*(t)* for each collected SAXS pattern, as a function of experimental time (*t*) is displayed in Figure 1d. The temporal changes of *Q*_*In*_*(t)*, that is independent of concentration, provide important insight into the structural evolution of the aqueous solution,^30,31^ though a large variation was spotted at the end of the measurement due to an instability of the shrinking droplet. The time-dependent co-assembly process was divided into five regions ***t***_***1***_***–t***_***5***_ that are related to different nanostructures that form with increasing concentration, Figure 1d.

The POM-2C_12_ and P123 solution was studied at a starting molar concentration of 12.0 mM (*ca*. 5.02 wt %), which lies in the ***t***_***1***_ region and corresponds to the red SAXS patterns in Figure 1e. Dynamic light scattering (DLS) (Figure S3) and cryo-TEM (Figure 2a) indicate that the dilute solution contains vesicles with sizes ranging between 235 and 265 nm. The SAXS pattern of the solution at 12.0 mM (Figure 2b) could be fitted to a random lamellar phase using SasView software (version 5.05), with a hydrophobic and hydrophilic slab at 35.2 ± 0.2 Å and 10.2 ± 3.5 Å, respectively. This indicates that the vesicles that formed in the diluted concentration range have a bilayer thickness of *ca*. 45.2 ± 3.5 Å, as illustrated in Figure S4. The zeta potential of the vesicles was determined to be −16.9 ± 3.2 eV, Figure S5, which is in between the zeta potential of the 12.0 mM POM-2C_12_ aqueous solution (−80.0 ± 6.0 eV) and the zeta potential of a 12.0 mM P123 aqueous solution, that as expected is very close to zero (0.34 ± 0.18 eV). The weak correlation peak (indicated by the arrow in Figure 1e and Figure S6) that appeared in the SAXS pattern from *ca*. 200 s (which corresponds to a concentration of 20.1 mM) at *ca*. 0.0183 Å^–1^ suggests a *d* spacing of about 340 Å between adjacent vesicles.

**Figure 2:**
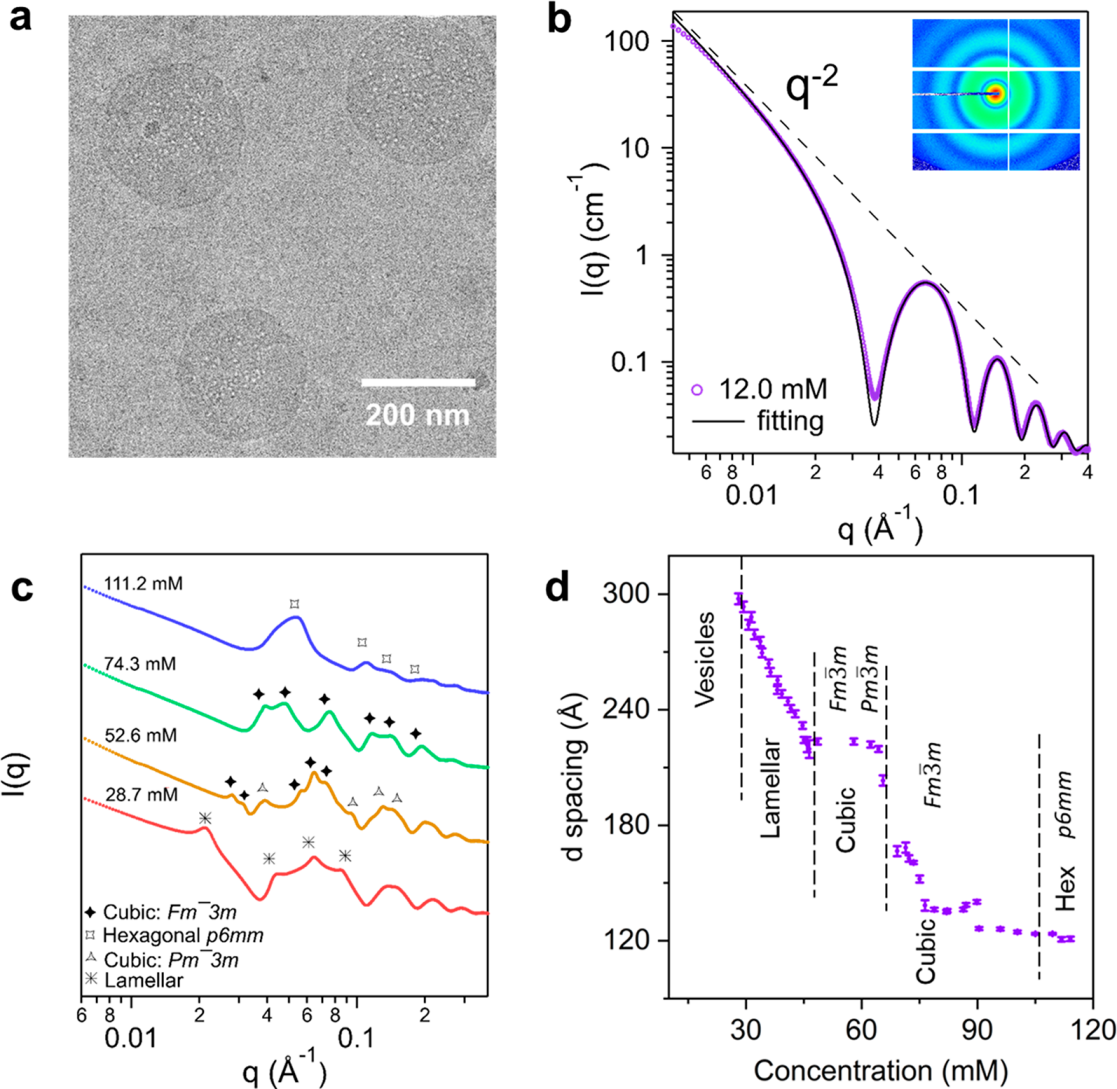
Structural characteristics of nanostructures formed in POM-2C_12_/P123 aqueous solutions with the mixing ratio 1:3. (a) Cryo-TEM image of vesicles from a dilute solution (2.4 mM). (b) SAXS pattern of a solution at a concentration of 12.0 mM and its corresponding fit into the random lamellar phase model. The corresponding fitting parameters are listed in Table S1. The inset is the 2D SAXS pattern. (c) SAXS patterns of solutions at four different concentrations with identified nanostructures. The SAXS patterns are offset vertically for clarity. (d) *d* spacings extracted from the main peaks of the time-resolved SAXS patterns, plotted as a function of molar concentration. The dashed lines represent estimated phase boundaries.

The SAXS pattern displayed several distinct peaks from 368 s, which related to a lamellar phase (Figure 1e and Figure 2c). This transformation corresponds to the ***t***_***2***_ region (Figure 1d) with an onset at a concentration of 28.7 mM. The correlation peak shifted to higher *q* with time, Figure S7, between 368 and 638 s, which indicates the formation of a more closely packed lamellar phase with increasing concentration.

At concentrations of 52.6 mM and above (from 638 s, related to the region ***t***_***3***_ in Figure 1d), the SAXS pattern corresponded to a mixed cubic phase, consisting of the *Fm*3̅*m* space group and the *Pm*3̅*m* space group, Figure 2c. The Bonnet ratio (the ratio of the lattice parameters of the two coexisting cubic phases, a_*Fm*3*m*_/a_*Pm*3*m*_)^32−34^ was calculated for the concentration at 52.6 mM, as listed in Table S2. As the droplet continued to shrink and the concentration thus increased, the SAXS analysis suggested that the nanostructures in the droplet transform to a single cubic phase with the *Fm*3̅*m* space group from 74.3 mM (***t***_***4***_ region in Figure 1d), followed by a final phase transformation into a hexagonal phase (***t***_***5***_ region) toward the end of the measurement, at 111.2 mM (Figure 1d).

The large vesicles that formed in the dilute concentration range in the POM-2C_12_/P123 = 1:3 system can be attributed to the phase behavior of P123.^35^ The transition from vesicles to lamellar sheets with increasing concentration (indicated by an arrow in Figure 1e and further illustrated in Figure S8) is caused by the interactions between the overcrowded vesicles.^36^ The subsequent cubic phase existed at higher concentrations (to 106 mM) compared to a previously reported pure P123 system (to 93 mM at 20 °C).^37^ The difference might be due to the large size/high charge of the POM headgroups of POM-2C_12_ in the system, which may delay the subsequent formation of the hexagonal phase.

The *d* spacing decreased gradually with increasing concentration (Figure 2d). The most pronounced decrease of the *d* spacing occurred within the lamellar region where the distance between two adjacent bilayers experienced a decrease from *ca*. 296 Å to *ca*. 220 Å as the concentration increased from 28.7 mM to 45.9 mM. Between 47.0 and 65.0 mM, the *d* spacing remained almost unchanged at the beginning and then decreased slightly. The analysis of the *d* spacing plotted in Figure 2d that was calculated according to the first primary peak of the *Fm*3̅*m* space group was complicated by the existence of two cubic phases. The estimated *d* spacing decreased as only a single cubic phase with the *Fm*3̅*m* space group was formed (***t***_***4***_).

The time-resolved WAXS patterns of the POM-2C_12_/P123 system (Figure S9) showed that the crystal structure of the phosphotungstate headgroup remained unchanged throughout the measurement. This indicates that the POM headgroup is unaffected by the concentration increase and the X-ray beam. The dried beads that were collected from the levitator after the SAXS measurements of the POM-2C_12_/P123 (1:3) system have an ellipsoid shape with a cross-sectional size of 646 μm × 721 μm (Figure S10a). The rough surface of the dry bead (Figure S10b) is probably related to the drying-induced shrinkage. The EDS analyses (Figure S11) showed the existence of P, W, Si, C, K and O elements in the dry bead, which are the main components of the two molecules we have studied.

The SAXS measurements confirm that the dilute POM-2C_12_ solution (12.0 mM) contains ellipsoid micelles (Figure S12) similar to previous work where the POM-containing micelles were used as the template to encapsulate POMs in porous carriers.^38^ Interestingly, no ordered structures were observed at higher concentrations (between *ca*. 18.7 mM and 94.7 mM) in POM-2C_12_ solutions, as illustrated in Figure S12a. The scattering invariant (Figure S12b) displayed a continuous increase, which corroborated that no phase transition occurred within the investigated concentration range. We speculate that the high charge (refer to the zeta potential in Figure S5) and the large size of the hydrophilic headgroup^39^ of the POM-2C_12_ molecules impeded the organization of molecules even at high concentrations. Drying of the POM-2C_12_ solution on a glass slide, Figure S13, resulted in large rod-like deposits with a size of *ca*. 1.5 μm, similar to what has been found for a modified lacunary polyoxometalate cluster ([PW_11_O_39_]).^40^

We have also investigated the structural evolution of POM-2C_12_-rich mixed solutions with a mixing ratio between POM-2C_12_ and P123 of 3:1, Figure 3a–c. The POM-2C_12_-rich mixed solution, with a concentration of 12.0 mM, generated a SAXS pattern that could be fitted to elliptical cylinders^41^ with a 25.0 ± 0.5 Å radius and an axial ratio of 3.4 (Figure 3a) in SasView software (version 5.05). Fitting of DLS data using the CONTIN method resulted in a hydrodynamic size of *ca*. 100 Å with a polydispersity of 0.35; see the inset of Figure 3a. A representative cryo-TEM image, Figure 3b, corroborated the elliptical morphology. The formation of ellipsoids was shown in the POM-2C_12_/P123 = 3:1 system at 12.0 mM while the POM-2C_12_/P123 = 1:3 system was dominated by vesicles at similar concentrations, which could be related to a higher headgroup area due to a higher charge density for the system that contains more POM-2C_12_.^36^ This leads to a lower value of the packing parameter of the POM-2C_12_/P123 = 3:1 mixture that may reach under 1/3, a value range that predicts the formation of ellipsoids or spheres.^42^ Time-resolved SAXS measurements of the shrinking droplets of the POM-2C_12_-rich mixed solution, Figure 3c, showed one sharp peak and one broad peak (indicated by the arrows) at the end, which is related to a wormlike structure. No additional ordered structures or phase transitions could be detected up to the highest investigated concentration of 102.9 mM (Figure S14).

**Figure 3:**
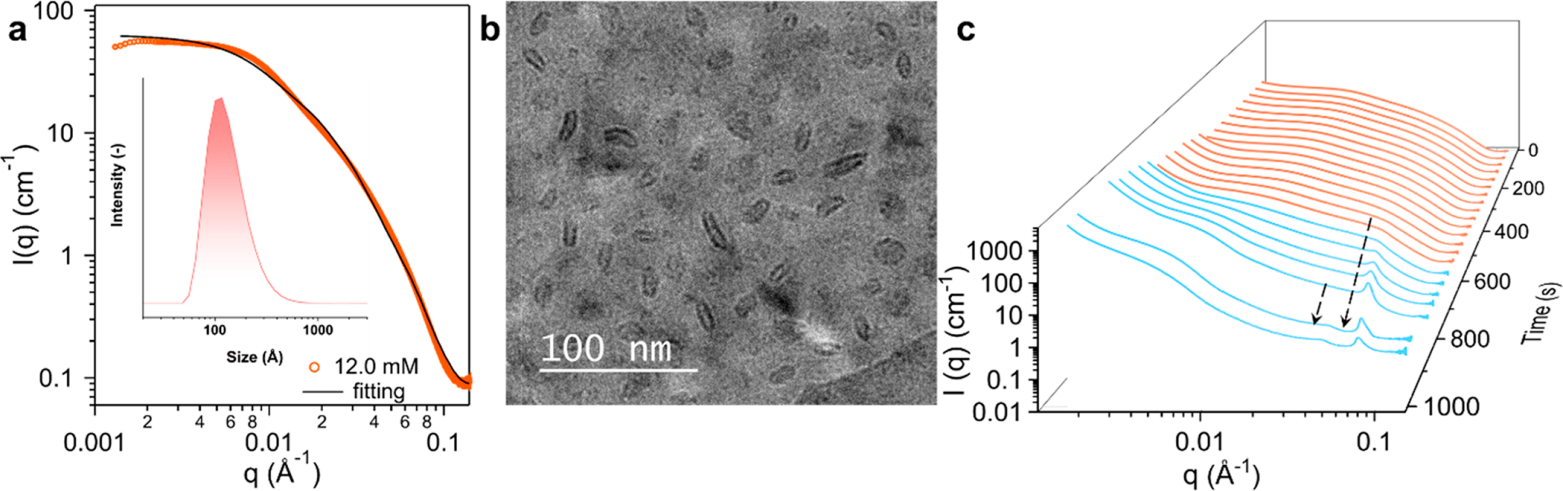
Structural characteristics of nanostructures formed in POM-2C_12_/P123 aqueous solutions with the mixing ratio 3:1. (a) SAXS pattern of a solution at the concentration of 12.0 mM and its corresponding fit into the elliptical cylinder model. The corresponding fitting parameters are listed in Table S3. The inset shows the size distribution determined by DLS. (b) Cryo-TEM image at the concentration of 12.0 mM. (c) Time-resolved SAXS patterns (*I(q)* versus *q*). The pink patterns and blue patterns correspond to ellipsoidal and wormlike phases, respectively. The two arrows indicate the positions where the two primary peaks are.

Mixing POM-2C_12_ with small nonionic molecules, *e.g.* octaethylene glycol monododecyl ether (C_12_EO_8_), resulted in the formation of disordered micelles at a concentration of 80.0 mM (see the brown curve in Figure S15). Increasing the concentration to 133.0 mM, resulted in the formation of an ordered lamellar phase (see the green curve in Figure S15).

These comparative studies clearly show that co-assembly of the covalently organo-functionalized POM clusters with the amphiphilic P123 block copolymer is responsible for the rich phase behavior in aqueous solutions.

The morphologies of the nanostructures formed in POM-2C_12_/P123 systems with mixing ratios of both 1:3 and 3:1 were investigated by dissipative particle dynamics (DPD) simulations at total molar concentrations where different phases were observed experimentally. The molecular structures and coarse-grained models are shown in Figure 4a.

**Figure 4:**
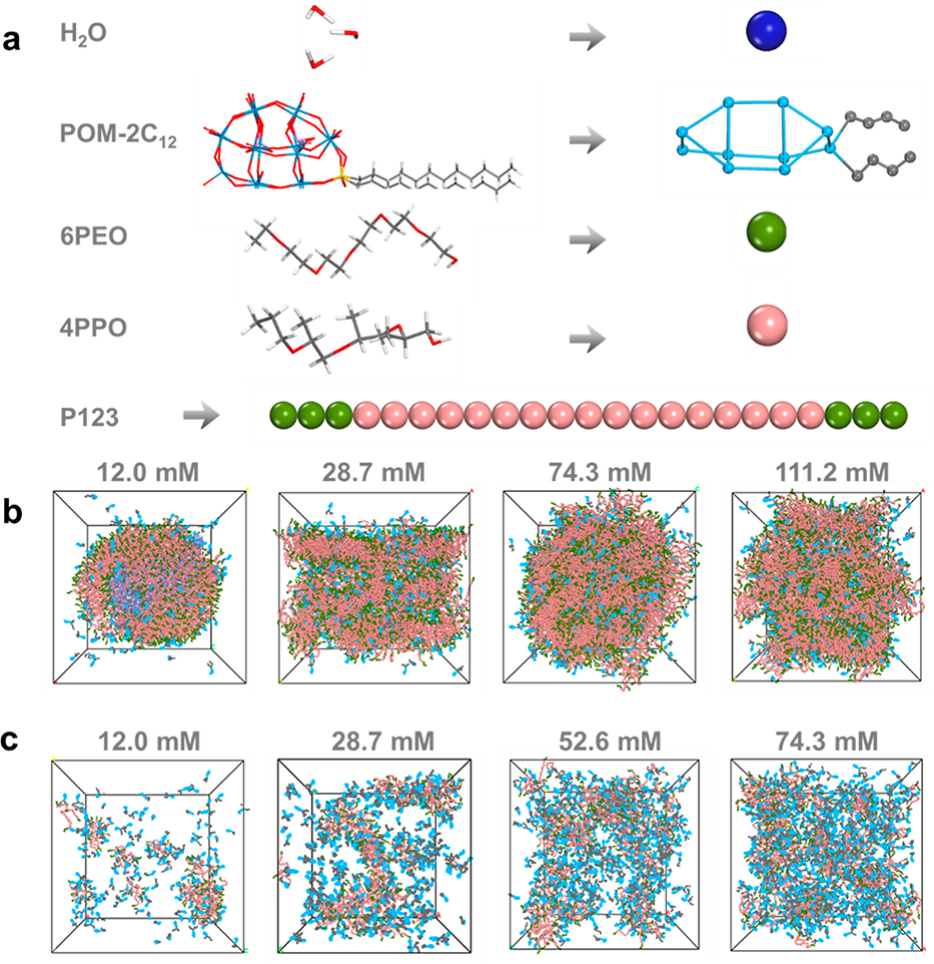
DPD simulations of the co-assembly of POM-2C_12_ and P123 in aqueous solutions. (a) Coarse-grained mapping of the molecules in this study. Representative snapshots from simulations on the nanostructures formed in aqueous solutions of different concentrations of: (b) POM-2C_12_/P123, mixing ratio 1:3, and (c) POM-2C_12_/P123, mixing ratio 3:1. The water molecules, except the water molecules in the inner core of the vesicles at the concentration of 12.0 mM, are omitted for clarity.

The DPD simulations for the POM-2C_12_/P123 system with a mixing ratio of 1:3 at a concentration of 12.0 mM (Figure 4b and Figure S16a) show large, spherical assembles that correspond well to the large vesicles that were observed experimentally at this concentration. The DPD simulations at increasing concentration (28.7 mM) also corroborate the experimental observation and indicate the formation of lamellar phase structures. The DPD simulations for the mixture with a concentration of 74.3 mM show a face-centered structure, where the SAXS study indicated that a cubic phase with the *Fm*3̅*m* space group is formed. At the highest simulated concentration (111.2 mM), the DPD simulation reproduce a hexagonal packing structure.

The morphologies of the nanostructures formed in the POM-2C_12_/P123 system with a mixing ratio of 3:1 (Figure 4c) are difficult to define quantitatively, but the DPD simulations appear to reproduce elliptical nanostructures at 12.0 mM, which corroborate with both the SAXS (Figure 3a) and cryo-TEM (Figure 3b) analyses. Simulations of solutions at a higher concentration (28.7, 52.6, and 74.3 mM) (Figure 4c, Figure S16b, Figure S17) indicated the formation of wormlike nanostructures.

Overall, the simulated structures follow the general trends that were inferred from the time-resolved SAXS data, but more detailed analyses of, *e.g.*, the region where two cubic space groups were found to coexist probably require that larger systems are used for simulations. The simulations clearly illustrate that the covalent organo-functionalization is able to minimize aggregation and promote the formation of ordered POM-containing nanostructures in aqueous solutions.

We have investigated the nanostructures formed by co-assembly of POM-2C_12_ and P123 in levitating aqueous droplets by time-resolved SAXS combined with dissipative particle dynamic simulations and cryo-TEM. The time-resolved SAXS measurements were performed on evaporating and thus shrinking levitating droplets that enabled studies of the structural evolution for concentrations ranging between 12.0 mM and 133.0 mM. The mixtures of POM-2C_12_ and P123 with a 1:3 ratio displayed a rich phase behavior with the formation of large vesicles at the lowest concentration (12.0 mM) that transformed into a lamellar phase, a mixture of two cubic phases that evolved into one dominating cubic phase, and eventually the formation of a hexagonal phase at the highest investigated concentration. The high electron density of POMs promoted the determination of nanostructures formed by the covalent-modified amphiphilic polyoxometalates in the shrinking levitating droplet and ensured a sufficient contrast of the cryo-TEM images.

## Methods

Small/wide angle X-ray scattering experiments were carried out at the ID02 beamline,^43^ European Synchrotron Radiation Facility (ESRF), Grenoble, France, using a standard X-ray energy of 12.3 keV. 2D SAXS patterns were collected using an Eiger 2-4 M detector using a sample-to-detector distance at 1.5, 3, or 5 m depending on the pretest of the size of the colloid in the solution. 2D WAXS patterns were collected using a Rayonix LX 170HS CCD camera at a sample to detector distance of 13 cm. The online data reduction pipeline is based on *Python Fast Azimuthal Integration* (*PyFAI*).^44^ The 2D SAXS/WAXS images were background-corrected and converted to 1D data by azimuthal integration of the 2D signal, and the obtained 1D data are visualized via SAXSutilities2 software,^45^ where *q* is defined by *q = 4π/λ* sin(*θ*/2) with the wavelength (λ) and the scattering angle θ. We used a customized setup to measure the self-assembled nanostructures while the concentration increased. An acoustic levitator, with a microscope camera in place to follow the status of the levitating droplet, was placed in positions as schematically depicted in Figure 1a. At the beginning of the measurement, a droplet of the aqueous solution (total surfactant molar concentration, 12.0 mM) was injected into the acoustic levitator (model 13K11, tec5, Oberursel, Germany) using microliter syringes (Hamilton Company, USA). The size of the droplet in the acoustic levitator was followed by the microscope camera. Videos that capture the shrinking process of the droplet were converted to image sequences by VirtualDub software. The size of the shrinking droplet with time was analyzed using ImageJ software and calibrated using the height scan at the beginning of each time-resolved SAXS measurement. For absolute intensity calibration and solvent background subtraction, scattering of the empty levitator chamber and levitator chamber with a water droplet were also recorded. Each experiment was repeated 2–3 times.

## Supporting Information Available

Molecular structure and synthesis of POM-2C_12_; preparation of the original solutions; NMR spectrum of POM-2C_12_; DLS result for the POM-2C_12_ solution; illustration of the vesicle structure; zeta potentials of aqueous solutions; SAXS pattern of POM-2C_12_/P123 mixtures; time-resolved SAXS patterns of levitating droplet containing POM-2C_12_/P123 mixtures; structural features of the SAXS patterns; time-resolved WAXS patterns of levitating droplet containing POM-2C_12_/P123 mixtures; SEM images of the dried bead; EDS results of the dried bead; time-resolved SAXS patterns of levitating droplet containing POM-2C_12_ and the relevant change of volume and scattering invariant; microscope image of drying solution containing POM-2C_12_; change of volume and scattering invariant of levitating droplet containing POM-2C_12_/P123; SAXS patterns of POM-2C_12_/C_12_EO_8_ mixtures; representative snapshots of DPD simulations results; and DPD simulations (PDF)

## References

[ref1] Liu Y.; Wu X.; Li Z.; Zhang J.; Liu S.-X.; Liu S.; Gu L.; Zheng L. R.; Li J.; Wang D. Fabricating Polyoxometalates-Stabilized Single-Atom Site Catalysts in Confined Space with Enhanced Activity for Alkynes Diboration. Nat. Commun. 2021, 12 (1), 1–9. 10.1038/s41467-021-24513-x. 34244508 PMC8271022

[ref2] Liu R.; Streb C. Polyoxometalate-Single Atom Catalysts (POM-SACs) in Energy Research and Catalysis. Adv. Energy Mater. 2021, 11 (25), 2101120 10.1002/aenm.202101120.

[ref3] Lei J.; Fan X.-X.; Liu T.; Xu P.; Hou Q.; Li K.; Yuan R.-M.; Zheng M.-S.; Dong Q.-F.; Chen J.-J. Single-Dispersed Polyoxometalate Clusters Embedded on Multilayer Graphene as a Bifunctional Electrocatalyst for Efficient Li-S Batteries. Nat. Commun. 2022, 13 (1), 1–10. 10.1038/s41467-021-27866-5. 35017484 PMC8752791

[ref4] Roger I.; Shipman M. A.; Symes M. D. Earth-Abundant Catalysts for Electrochemical and Photoelectrochemical Water Splitting. Nat. Rev. Chem. 2017, 1 (1), 1–13. 10.1038/s41570-016-0003.

[ref5] Wei H.; Huang K.; Wang D.; Zhang R.; Ge B.; Ma J.; Wen B.; Zhang S.; Li Q.; Lei M. Iced Photochemical Reduction to Synthesize Atomically Dispersed Metals by Suppressing Nanocrystal Growth. Nat. Commun. 2017, 8 (1), 1–8. 10.1038/s41467-017-01521-4. 29133795 PMC5684195

[ref6] Lee C.; Jeon D.; Park J.; Lee W.; Park J.; Kang S. J.; Kim Y.; Ryu J. Tetraruthenium Polyoxometalate as an Atom-Efficient Bifunctional Oxygen Evolution Reaction/Oxygen Reduction Reaction Catalyst and Its Application in Seawater Batteries. ACS Appl. Mater. Interfaces 2020, 12 (29), 32689–32697. 10.1021/acsami.0c07225. 32589016

[ref7] Ouyang M.; Papanikolaou K. G.; Boubnov A.; Hoffman A. S.; Giannakakis G.; Bare S. R.; Stamatakis M.; Flytzani-Stephanopoulos M.; Sykes E. C. H. Directing Reaction Pathways via in Situ Control of Active Site Geometries in PdAu Single-Atom Alloy Catalysts. Nat. Commun. 2021, 12 (1), 1–11. 10.1038/s41467-021-21555-z. 33750788 PMC7943817

[ref8] He P.; Xu B.; Xu X.; Song L.; Wang X. Surfactant Encapsulated Palladium-Polyoxometalates: Controlled Assembly and Their Application as Single-Atom Catalysts. Chem. Sci. 2016, 7 (2), 1011–1015. 10.1039/C5SC03554F. 29896369 PMC5954849

[ref9] Isono T.; Komaki R.; Lee C.; Kawakami N.; Ree B. J.; Watanabe K.; Yoshida K.; Mamiya H.; Yamamoto T.; Borsali R. Rapid Access to Discrete and Monodisperse Block Co-Oligomers from Sugar and Terpenoid toward Ultrasmall Periodic Nanostructures. Commun. Chem. 2020, 3 (1), 1–9. 10.1038/s42004-020-00385-y. 36703322 PMC9814839

[ref10] Dick B.; Sit J. C.; Brett M. J.; Votte I. M. N.; Bastiaansen C. W. M. Embossed Polymeric Relief Structures as a Template for the Growth of Periodic Inorganic Microstructures. Nano Lett. 2001, 1 (2), 71–73. 10.1021/nl0055153.

[ref11] Genov D. A.; Sarychev A. K.; Shalaev V. M.; Wei A. Resonant Field Enhancements from Metal Nanoparticle Arrays. Nano Lett. 2004, 4 (1), 153–158. 10.1021/nl0343710.

[ref12] Rybczynski J.; Banerjee D.; Kosiorek A.; Giersig M.; Ren Z. F. Formation of Super Arrays of Periodic Nanoparticles and Aligned ZnO Nanorods– Simulation and Experiments. Nano Lett. 2004, 4 (10), 2037–2040. 10.1021/nl048763y.

[ref13] Kapuscinski M.; Agthe M.; Lv Z.-P.; Liu Y.; Segad M.; Bergström L. Temporal Evolution of Superlattice Contraction and Defect-Induced Strain Anisotropy in Mesocrystals during Nanocube Self-Assembly. ACS Nano 2020, 14 (5), 5337–5347. 10.1021/acsnano.9b07820. 32338498 PMC7343289

[ref14] Nisar A.; Wang X. Surfactant-Encapsulated Polyoxometalate Building Blocks: Controlled Assembly and Their Catalytic Properties. Dalt. Trans. 2012, 41 (33), 9832–9845. 10.1039/c2dt30470h. 22801936

[ref15] Yang Y.; Zhang B.; Wang Y.; Yue L.; Li W.; Wu L. A Photo-Driven Polyoxometalate Complex Shuttle and Its Homogeneous Catalysis and Heterogeneous Separation. J. Am. Chem. Soc. 2013, 135 (39), 14500–14503. 10.1021/ja4057882. 24041266

[ref16] Yin S.; Sun H.; Yan Y.; Li W.; Wu L. Hydrogen-Bonding-Induced Supramolecular Liquid Crystals and Luminescent Properties of Europium-Substituted Polyoxometalate Hybrids. J. Phys. Chem. B 2009, 113 (8), 2355–2364. 10.1021/jp810262c. 19193055

[ref17] Nisar A.; Lu Y.; Zhuang J.; Wang X. Polyoxometalate Nanocone Nanoreactors: Magnetic Manipulation and Enhanced Catalytic Performance. Angew. Chemie Int. Ed. 2011, 50 (14), 3187–3192. 10.1002/anie.201006155. 21370361

[ref18] Nisar A.; Zhuang J.; Wang X. Construction of Amphiphilic Polyoxometalate Mesostructures as a Highly Efficient Desulfurization Catalyst. Adv. Mater. 2011, 23 (9), 1130–1135. 10.1002/adma.201003520. 21360767

[ref19] Lunkenbein T.; Kamperman M.; Li Z.; Bojer C.; Drechsler M.; Förster S.; Wiesner U.; Müller A. H. E.; Breu J. Direct Synthesis of Inverse Hexagonally Ordered Diblock Copolymer/Polyoxometalate Nanocomposite Films. J. Am. Chem. Soc. 2012, 134 (30), 12685–12692. 10.1021/ja304073t. 22757978

[ref20] Wang Y.; Wang X.; Zhang X.; Xia N.; Liu B.; Yang J.; Yu W.; Hu M.; Yang M.; Wang W. Manipulation of Ordered Nanostructures of Protonated Polyoxometalate through Covalently Bonded Modification. Chem.—Eur. J. 2010, 16 (42), 12545–12548. 10.1002/chem.201001674. 20853294

[ref21] Zhang J.; Song Y.-F.; Cronin L.; Liu T. Self-Assembly of Organic– Inorganic Hybrid Amphiphilic Surfactants with Large Polyoxometalates as Polar Head Groups. J. Am. Chem. Soc. 2008, 130 (44), 14408–14409. 10.1021/ja805644a. 18841899

[ref22] Liu Q.; Wang X. Polyoxometalate Clusters: Sub-Nanometer Building Blocks for Construction of Advanced Materials. Matter 2020, 2 (4), 816–841. 10.1016/j.matt.2020.01.020.

[ref23] Liu S.; Tang Z. Polyoxometalate-Based Functional Nanostructured Films: Current Progress and Future Prospects. Nano Today 2010, 5 (4), 267–281. 10.1016/j.nantod.2010.05.006.

[ref24] Landsmann S.; Lizandara-Pueyo C.; Polarz S. A New Class of Surfactants with Multinuclear, Inorganic Head Groups. J. Am. Chem. Soc. 2010, 132 (14), 5315–5321. 10.1021/ja1011178. 20302305

[ref25] Czakkel O.; Nagy B.; Geissler E.; László K. In Situ SAXS Investigation of Structural Changes in Soft Resorcinol–Formaldehyde Polymer Gels during CO_2_-Drying. J. Supercrit. Fluids 2013, 75, 112–119. 10.1016/j.supflu.2012.12.027.

[ref26] Stribeck N. X-Ray Scattering of Soft Matter; Springer Science & Business Media, 2007.

[ref27] Feigin L. A.; Svergun D. I. Structure Analysis by Small-Angle X-Ray and Neutron Scattering; Plenum Press: New York, 1987.

[ref28] Li T.; Senesi A. J.; Lee B. Small Angle X-Ray Scattering for Nanoparticle Research. Chem. Rev. 2016, 116 (18), 11128–11180. 10.1021/acs.chemrev.5b00690. 27054962

[ref29] Da Vela S.; Begam N.; Dyachok D.; Schäufele R. S.; Matsarskaia O.; Braun M. K.; Girelli A.; Ragulskaya A.; Mariani A.; Zhang F. Interplay between Glass Formation and Liquid–Liquid Phase Separation Revealed by the Scattering Invariant. J. Phys. Chem. Lett. 2020, 11 (17), 7273–7278. 10.1021/acs.jpclett.0c02110. 32787309

[ref30] Yi Z.; Dumée L. F.; Garvey C. J.; Feng C.; She F.; Rookes J. E.; Mudie S.; Cahill D. M.; Kong L. A New Insight into Growth Mechanism and Kinetics of Mesoporous Silica Nanoparticles by in Situ Small Angle X-Ray Scattering. Langmuir 2015, 31 (30), 8478–8487. 10.1021/acs.langmuir.5b01637. 26158700

[ref31] Beyer F. L.; Masser K. A.; Lenhart J. L. Application of the Small-angle Scattering Invariant to Morphological Behavior in Ballistic Materials. J. Appl. Polym. Sci. 2021, 138 (21), 50478 10.1002/app.50478.

[ref32] Tran N.; Mulet X.; Hawley A. M.; Conn C. E.; Zhai J.; Waddington L. J.; Drummond C. J. First Direct Observation of Stable Internally Ordered Janus Nanoparticles Created by Lipid Self-Assembly. Nano Lett. 2015, 15 (6), 4229–4233. 10.1021/acs.nanolett.5b01751. 25984944

[ref33] Verma G.; Aswal V. K.; Fritz-Popovski G.; Shah C. P.; Kumar M.; Hassan P. A. Dilution Induced Thickening in Hydrotrope-Rich Rod-like Micelles. J. Colloid Interface Sci. 2011, 359 (1), 163–170. 10.1016/j.jcis.2011.03.061. 21507419

[ref34] Larsson K.; Tiberg F. Periodic Minimal Surface Structures in Bicontinuous Lipid–Water Phases and Nanoparticles. Curr. Opin. Colloid Interface Sci. 2005, 9 (6), 365–369. 10.1016/j.cocis.2004.12.002.

[ref35] Calori I. R.; Pinheiro L.; Braga G.; de Morais F. A. P.; Caetano W.; Tedesco A. C.; Hioka N. Interaction of Triblock Copolymers (Pluronic®) with DMPC Vesicles: A Photophysical and Computational Study. Spectrochim. Acta Part A Mol. Biomol. Spectrosc. 2022, 275, 121178 10.1016/j.saa.2022.121178. 35366523

[ref36] Bergström M.; Pedersen J. S.; Schurtenberger P.; Egelhaaf S. U. Small-Angle Neutron Scattering (SANS) Study of Vesicles and Lamellar Sheets Formed from Mixtures of an Anionic and a Cationic Surfactant. J. Phys. Chem. B 1999, 103 (45), 9888–9897. 10.1021/jp991846w.

[ref37] Wanka G.; Hoffmann H.; Ulbricht W. Phase Diagrams and Aggregation Behavior of Poly (Oxyethylene)-Poly (Oxypropylene)-Poly (Oxyethylene) Triblock Copolymers in Aqueous Solutions. Macromolecules 1994, 27 (15), 4145–4159. 10.1021/ma00093a016.

[ref38] Di A.; Schmitt J.; da Silva M. A.; Hossain K. M. Z.; Mahmoudi N.; Errington R. J.; Edler K. J. Self-Assembly of Amphiphilic Polyoxometalates for the Preparation of Mesoporous Polyoxometalate-Titania Catalysts. Nanoscale 2020, 12 (43), 22245–22257. 10.1039/D0NR05967F. 33141144

[ref39] Mitchell S. G.; de la Fuente J. M. The Synergistic Behavior of Polyoxometalates and Metal Nanoparticles: From Synthetic Approaches to Functional Nanohybrid Materials. J. Mater. Chem. 2012, 22 (35), 18091–18100. 10.1039/c2jm33128d.

[ref40] Klaiber A.; Polarz S. Passing Current through Electrically Conducting Lyotropic Liquid Crystals and Micelles Assembled from Hybrid Surfactants with π-Conjugated Tail and Polyoxometalate Head. ACS Nano 2016, 10 (11), 10041–10048. 10.1021/acsnano.6b04677. 27809472 PMC5235242

[ref41] Onsager L. The Effects of Shape on the Interaction of Colloidal Particles. Ann. N.Y. Acad. Sci. 1949, 51 (4), 627–659. 10.1111/j.1749-6632.1949.tb27296.x.

[ref42] Chen S.; Yang B.; Guo C.; Ma J.-H.; Yang L.-R.; Liang X.; Hua C.; Liu H.-Z. Spontaneous Vesicle Formation of Poly (Ethylene Oxide)– Poly (Propylene Oxide)– Poly (Ethylene Oxide) Triblock Copolymer. J. Phys. Chem. B 2008, 112 (49), 15659–15665. 10.1021/jp8019039. 19367948

[ref43] Narayanan T.; Sztucki M.; Zinn T.; Kieffer J.; Homs-Puron A.; Gorini J.; Van Vaerenbergh P.; Boesecke P. Performance of the Time-Resolved Ultra-Small-Angle X-Ray Scattering Beamline with the Extremely Brilliant Source. J. Appl. Crystallogr. 2022, 55 (1), 98–111. 10.1107/S1600576721012693. 35145357 PMC8805168

[ref44] Kieffer J.; Karkoulis D. PyFAI, a Versatile Library for Azimuthal Regrouping. In Journal of Physics: Conference Series; IOP Publishing, 2013; Vol. 425, p 202012.

[ref45] Sztucki M. SAXSutilities2: A Graphical User Interface for Processing and Analysis of Small-Angle X-Ray Scattering Data; 2021. 10.5281/zenodo.5825707.

