# Supplementary material for: Tunable Ordered Nanostructured Phases by Co-assembly of Amphiphilic Polyoxometalates and Pluronic Block Copolymers
Source: Nano Lett. 2023 Feb 16;23(5):1645–51. doi: 10.1021/acs.nanolett.2c03068 (PMC9999449; doi:10.1021/acs.nanolett.2c03068)
Supplement: Supplementary file 1 — nl2c03068_si_001.pdf [file nl2c03068_si_001.pdf]

SUPPLEMENTARY INFORMATION

Tunable Ordered Nanostructured Phases by Co-assembly of Amphiphilic Polyoxometalates and Pluronic Block Copolymers

Andi Di[1], Jipeng Xu[2], Thomas Zinn[3], Michael Sztucki[3], Wentao Deng[4], Anumol Ashok[1], Cheng Lian[2] and Lennart Bergström[1]**

1 Department of Materials and Environmental Chemistry, Stockholm University, Stockholm, 106 91, Sweden.

2 School of Chemistry and Molecular Engineering, East China University of Science and Technology, Shanghai, 200231, China

3 ESRF-European Synchrotron Radiation Facility, 38043 Grenoble, France

4 College of Chemistry and Chemical Engineering, Central South University, Changsha, 410083, China

Experimental details

Materials

$K_6[P_2W_{17}OSi_2(C_{12}H_{25})_2\}]$ (POM-2C_{12}, n = 12) were synthesized according to a published protocol.[1] And the molecular structure of POM-2C_{12} is given in ESI Fig. 1 for illustration. Poly(ethylene glycol)-*block*-poly(propylene glycol)-*block*-poly(ethylene glycol) (P123, purity > 98%) was purchased from Sigma Aldrich and were used as supplied. All hydrogenated surfactant solutions were prepared in ultrapure water (18.2 MΩ·cm, from an ELGA PURELAB flex water purification system).

Synthesis of POM-2C_{12}

The synthesis of POM-2C_{12} includes three main steps: (A) the synthesis of $K_6[\alpha\text{-}P_2W_{18}O_{62}]\cdot 14H_2O$, (B) the synthesis of $K_{10}[P_2W_{17}O_{61}]\cdot 19H_2O$ and (C) the attachment of hydrocarbon chains.

(A) The Dawson structured polyoxometalate, $K_6[\alpha\text{-}P_2W_{18}O_{62}]\cdot 14H_2O$, was prepared according to a well-established method, described by Droege in 1984.[2] The synthesis includes five steps: (1) 250 grams (0.76 mols) $Na_2WO_4\cdot 2H_2O$ was fully dissolved in 500 ml water, followed by the addition of 210 ml (3.09 mols) orthophosphoric acid (85 wt.%). The solution was then refluxed for 4 hours. If any greenish appeared during the reflux, a few drops of bromine can be added to the system. (2) 100 grams of ammonium chloride was added to the solution after cooling down. The solution was kept stirring for 10 min. Then the pale green salt was obtained after filtration. (3) The obtained pale green salt was then added to 100 ml NH_4Cl (25 grams) aqueous solution, and the mixture was stirred for a few minutes and filtered to recover the pale green salt. (4) The pale green salt was then fully dissolved in 300 ml of hot water (80 °C), and the solution was then put in a 4 °C fridge. There might be needle crystals crystallized out from the solution after cooling down. The needle crystal was impurities and needed to be removed from the solution by filtration. (5) The $K_6[\alpha\text{-}P_2W_{18}O_{62}]\cdot 14H_2O$ precipitates were obtained after adding 25 grams KCl into the cooled solution in step (4).

(B) Air dried $K_6[\alpha\text{-}P_2W_{18}O_{62}]\cdot 14H_2O$ (80 grams, 0.0115 mols) was dissolved in 200 ml water, followed by the addition of $KHCO_3$ solution (20 grams in 200 ml water) under stirring. The reaction was left under stirring for 1 hour, and then filtered to get white precipitates. The obtained white precipitates was then dissolved in 500 ml hot water (95 °C) and then the solution was left to cool down to room temperature. The white crystals that precipitate from the solution are the $K_{10}[P_2W_{17}O_{61}]\cdot 19H_2O$ product.

(C) 5.11 grams of $K_{10}[P_2W_{17}O_{61}]\cdot 20H_2O$ was dispersed in 180 ml anhydrous acetonitrile under vigorous stirring for 4 hours. Then 2.05 grams of $C_{12}H_{25}SiCl_3$ diluted in anhydrous $CHCl_3$ was added into the dispersion dropwise in about an hour. The obtained solution was filtered to remove any insoluble impurities. And then the solution was rotary evaporated to obtain the $K_6[P_2W_{17}O_{61}\{OSi_2(C_{12}H_{25})_2\}]$, denoted as POM-2$C_{12}$.

The preparation of solution samples

50 mM poly(ethylene glycol)-block-poly(propylene glycol)-block-poly(ethylene glycol) (P123) stock solution in water was firstly prepared. For mixture solutions containing POM-$2C_{12}$ and P123, POM-$2C_{12}$ powder was weighed out and dissolved in milli-Q water, and then a specific amount of P123 stock solution was added to obtain POM-$2C_{12}$/P123 mixture solutions with different mixing ratios.

Calculations

The partial scattering invariant ($Q_{In}(t)$), as a shape/size/interaction-independent indicator of changes in pore volume fraction, for each small angle X-ray scattering pattern was calculated by integrating $I(q)$ in the 1D reduced data including a q^2 factor:

$$Q_{In}(t) = \frac{1}{2\pi^2} \int_{q_{min}}^{q_{max}} q^2 I(q) dq$$

$Q_{In}(t)$ quantifies the net scattering power of a sample.[3] And here we calculate the $Q_{In}(t)$ using integration limits of q = 0.00416 to 0.4472 $Å^{-1}$ using Sasview software (version 5.05), and is plotted as a function of experimental time (*t*).

Characterisations

Scanning electron microscopy (SEM) images were collected on a Tabletop Microscope TM3000 (Hitachi, Ltd. Japan) equipped with Bruker Quantax 70 EDS (resolution: 135eV). ^{31}P NMR was measured using a relaxation time of 20 seconds. Aqueous solutions were measured at 25 °C using dynamic light scattering (DLS) using a Malvern Zetasizer Nano ZSP (Malvern, UK). Samples were measured at a scattering angle of 173 ° and a wavelength of 632.8 nm for 120 seconds, repeated 5 times. The size distributions, weighted in volume, was extracted using the CONTIN method. The zeta-potential of the micelles was measured, at the same concentration, using the same instrument. The vitrification of Cryo transmission electron microscopy (cryo-TEM) sample was done using a Thermo Scientific Vitrobot system. 3 μl of the sample solution was placed on a glow discharged Quantifoil-Copper TEM grid and blotted for 3 seconds to obtain a thin aqueous film and plunge frozen in liquid ethane. The Vitrobot chamber was kept at 22 °C and 100 % relative humidity. Cryo-TEM was carried out on a JEOL JEM-2100 microscope with a Gatan 914 Cryo-transfer holder. The images were recorded using a Gatan Orius 200D camera.

Molecular structure of POM-2C_{12}

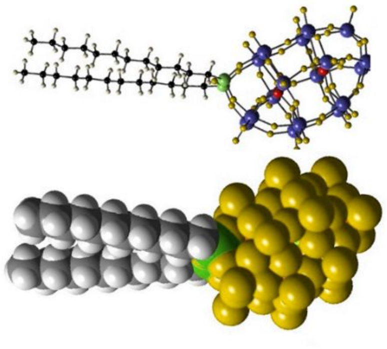

Figure S1. Ball-and-stick and space-filling representations of the structure of POM-2C_{12}, colour code: W (purple), P (red), O (yellow), Si (green), C (black) and H (white).

NMR spectrum of POM-2C_{12}

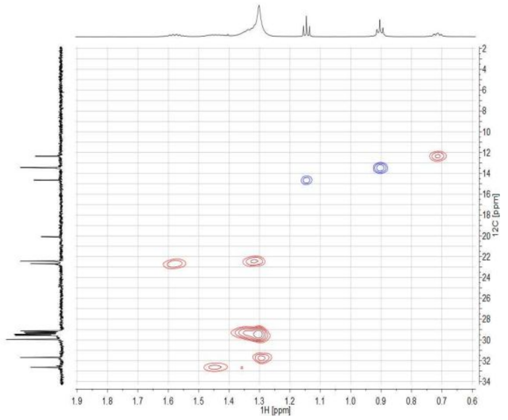

Figure S2. Carbon-hydrogen correlated NMR of POM-2C_{12}.

DLS result for POM-$2C_{12}$ solution

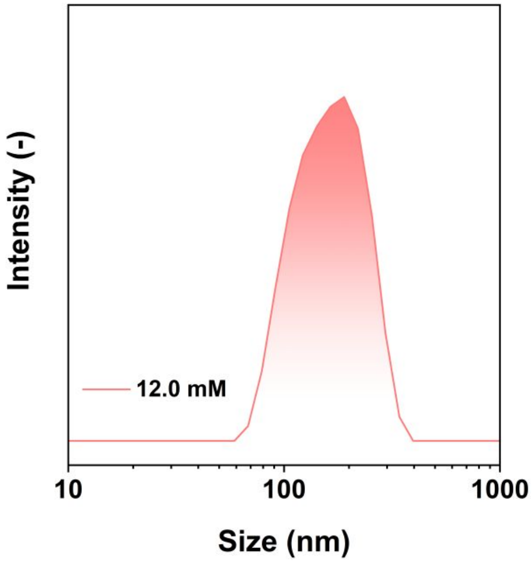

Figure S3. Volume-weighted size distribution of a 12.0 mM solution, obtained *via* the CONTIN method.

Fitting parameters of POM-$2C_{12}$/P123 = 1:3 mixture

Table S1. Fitted random lamellar phase model[4] parameters for nanostructures formed in 12.0 mM POM-$2C_{12}$/P123 aqueous solutions with a mixing ratio of 1:3.

Parameters							
Fitted parameters					Fixed parameters		
length_tail (Å)	length_head (Å)	sld_head ($\times 10^{-6}$ Å^{-2})	PD	scale	sld_tail ($\times 10^{-6}$ Å^{-2})	sld_solvent ($\times 10^{-6}$ Å^{-2})	Background (cm^{-1})
35.2 ± 0.2	10.2 ± 3.5	19.8 ± 0.1	0.35	0.010	9.5	9.47	0.015

*length_tail: tail thickness; length_head: head thickness; sld_tail: tail X-ray scattering length density (calculated); sld_head: head X-ray scattering length density; sld_solvent: solvent X-ray scattering length density; PD: polydispersity in length_head.

Illustration of vesicle structure

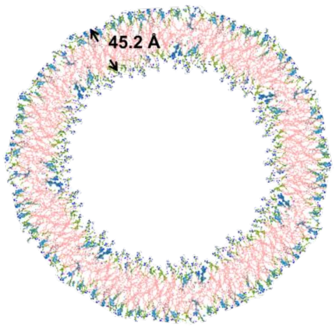

Figure S4. Illustration of the vesicle structure formed at 12.0 mM.

Zeta potentials of aqueous solutions

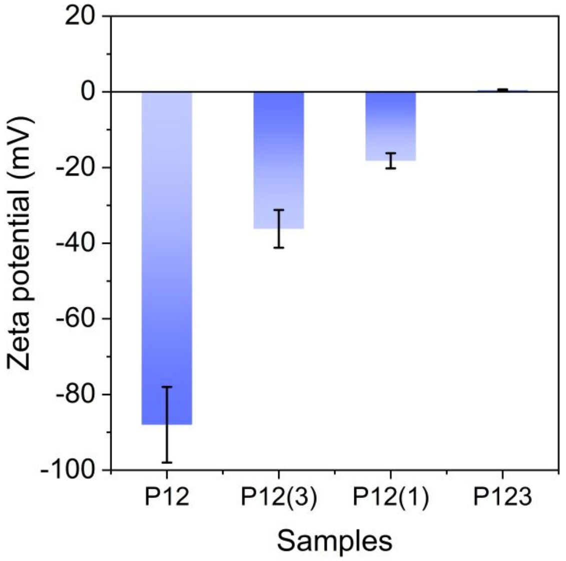

Figure S5. Zeta potentials of aqueous solutions, including sample P12 (12.0 mM POM-$2C_{12}$ soltion), P12(3) (12.0 mM POM-$2C_{12}$:P123 =3:1 solution), P12(1) (12.0 mM POM-$2C_{12}$:P123 =1:3 solution), and P123 (12.0 mM P123 solution).

SAXS pattern of POM-2C_{12}/P123 mixtures

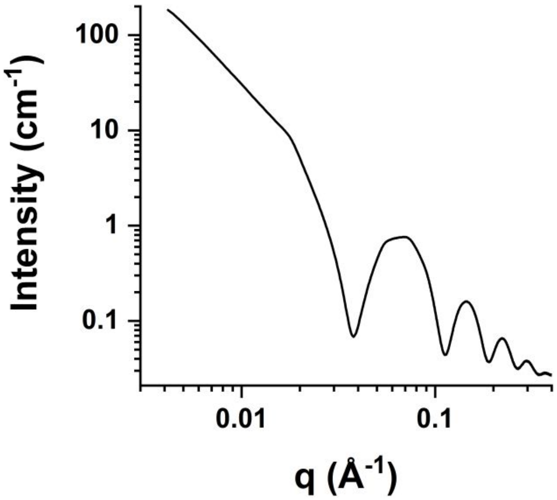

Figure S6. SAXS pattern of POM-2C_{12}/P123 (mixing ratio 1:3) system in the shrinking levitating droplet, correspond to the experimental time at 190 s in Fig. 1e.

Time-resolved SAXS patterns of levitating droplet contains POM-2C_{12}/P123 mixtures

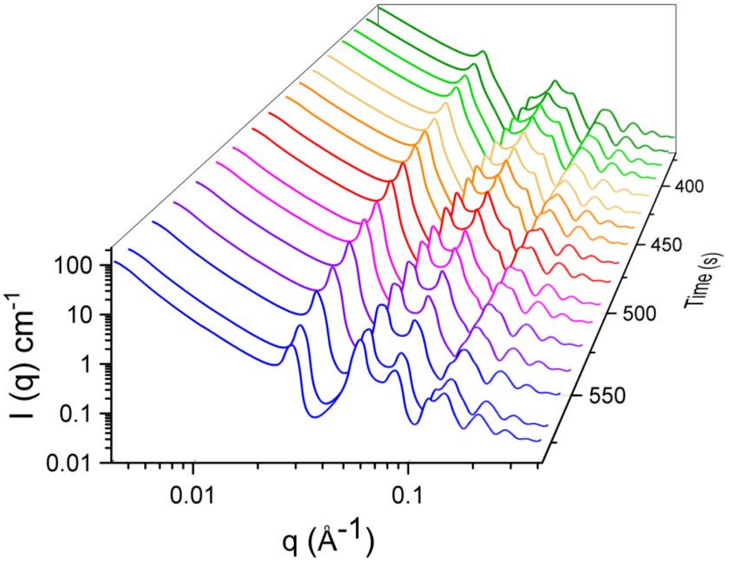

Figure S7. Time-resolved SAXS patterns between 368 and 584 s (concentration ranges between 28.7 and 45.9 mM).

Structure factor of the interaction vesicles

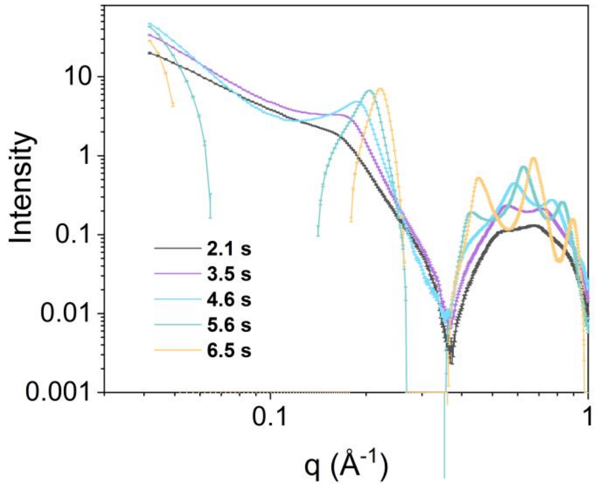

Figure S8. Structure factor that is directly related to the interactions between the vesicles. The structure factor was calculated by subtracting the form factor from SAXS patterns that have correlation peaks.

Structure features of the SAXS patterns

Table S2. Structure features at selected concentrations in the generated liquid crystalline phases (plotted in Fig. 2e).

Concentration	Space group	Lattice parameter a (Å)	Bonnet Ratio
28.7	Lamellar	299.0	--
52.6	$Fm\bar{3}m$, $Pm\bar{3}m$	160.0, 130.6	1.22

74.3	$Fm\bar{3}m$	161.0	--
111.2	*P6mm*	116.3	--

Fitting parameters of POM-2C_{12}/P123 = 3:1 mixture

Table S3. Fitted elliptical cylinder model[5] parameters for nanostructures formed in 12.0 mM POM-2C_{12}/P123 aqueous solutions with a mixing ratio 3:1.

Parameters						
Fitted parameters					Fixed parameters	
radius_minor (Å)	axis_ratio	Length (Å)	sld ($\times 10^{-6}$ Å^{-2})	scale	sld_solvent ($\times 10^{-6}$ Å^{-2})	Background (cm^{-1})
25.0 ± 0.5	3.4 ± 1.0	570 ± 10	16.6 ± 0.3	0.0032	9.47	0.08

* radius_minor: ellipse minor radius; axis_ratio: ratio of major radius over minor radius; length: length of the cylinder; sld: cylinder X-ray scattering length density; sld_head: head X-ray scattering length density; sld_solvent: solvent X-ray scattering length density.

Time-resolved WAXS patterns of levitating droplet contains POM-2C_{12}/P123 mixtures

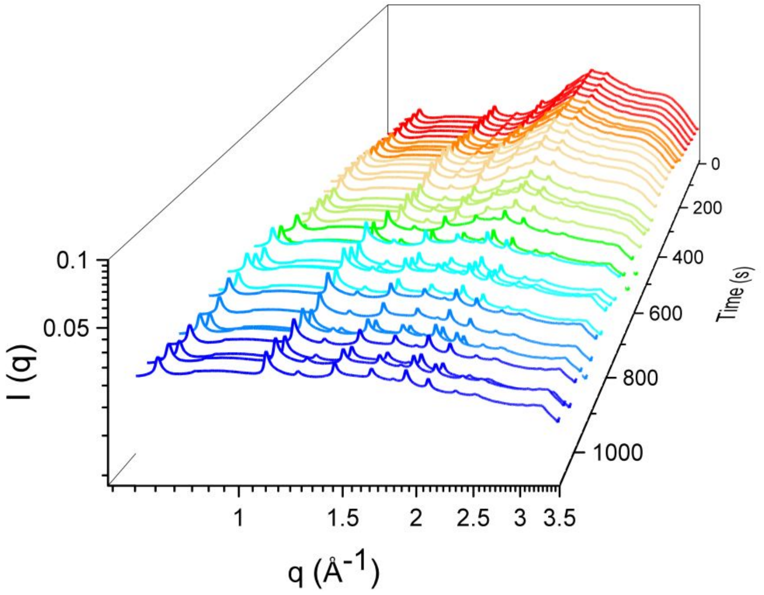

Figure S9. Time-resolved WAXS patterns of the POM-2C_{12}/P123 (1:3) aqueous drop.

SEM images of the dried bead

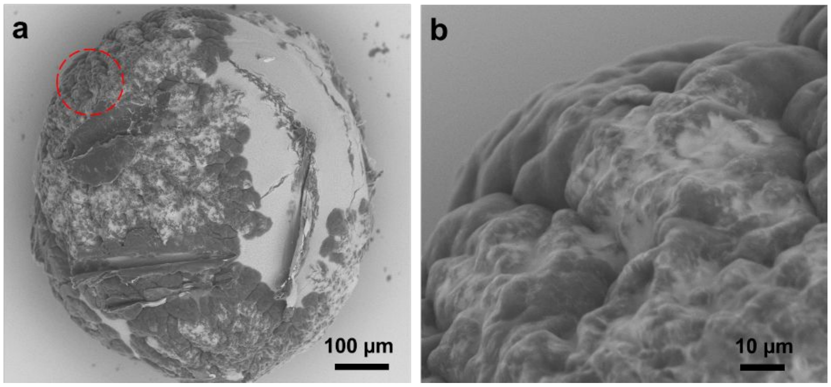

Figure S10. SEM images of dry beads of POM-2C_{12}:P123 =1:3 system. **a** The whole dry bead, and **b** a zoom from the region indicated with a red circle in **a**.

EDS results of the dried bead

a

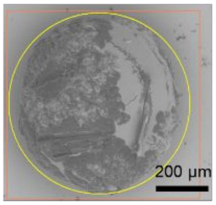

b

Spectrum: Point

Element	AN	Series	Net	unn. C [wt.%]	norm. C [wt.%]	Atom. C [at.%]	Error [%]
Carbon	6	K-series	90818	89.33	59.53	72.36	9.6
Oxygen	8	K-series	30565	29.05	19.36	17.67	3.3
Silicon	14	K-series	234076	28.11	18.73	9.74	1.2
Tungsten	74	L-series	1873	3.37	2.25	0.18	0.1
Potassium	19	K-series	854	0.17	0.11	0.04	0.0
Phosphorus	15	K-series	153	0.03	0.02	0.01	0.0
			Total:	150.06	100.00	100.00	

c

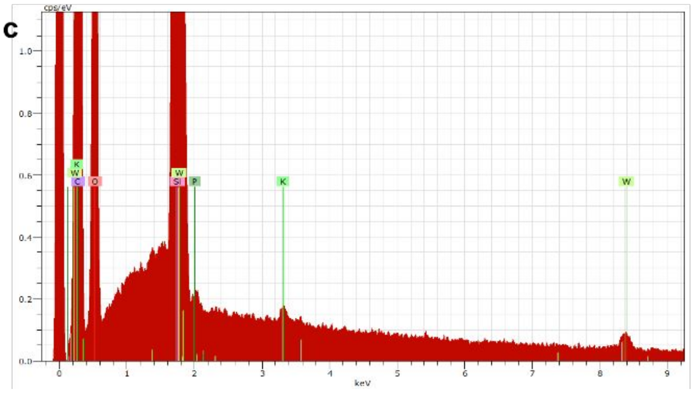

Figure S11. a SEM image of the whole dry bead with the area of EDS analysis indicated in a yellow circle, **b** EDS analysis results, and **c** EDS spectrum.

Time-resolved SAXS patterns of levitating droplet contains POM-2C_{12} and the relevant change of volume and scattering invariant

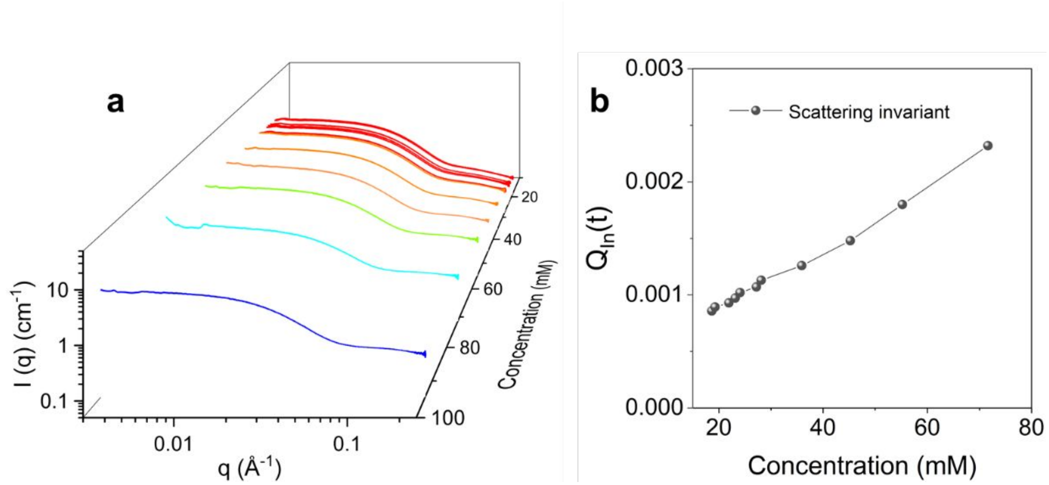

Figure S12. a Time resolved SAXS patterns of the shrinking droplet contains POM-2C_{12} with concentration ranges between 18.7 mM and 94.7 mM. **b** Change of scattering invariant with concentration.

Microscope image of drying solution contains POM-2C_{12}

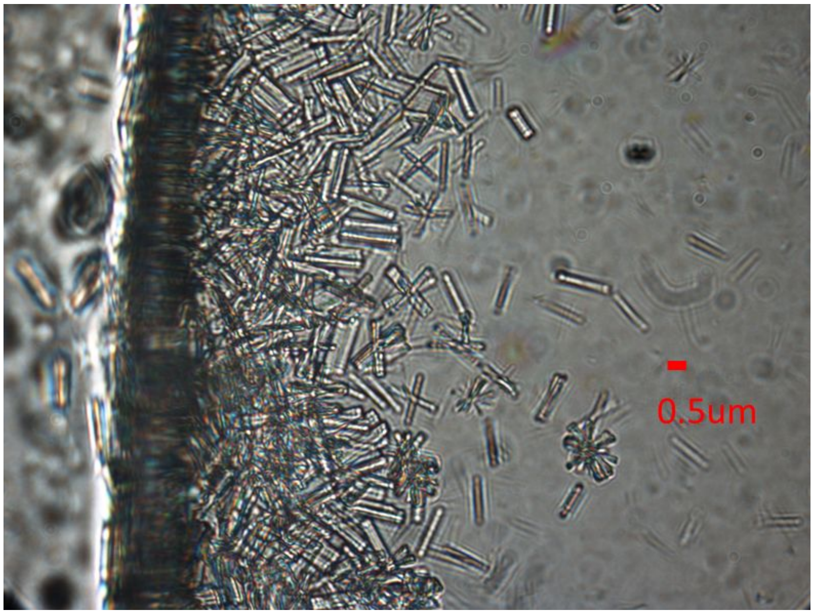

Figure S13. Microscope image of the dried POM-2C_{12} solution.

Change of volume and scattering invariant of levitating droplet contains POM-$2C_{12}$/P123

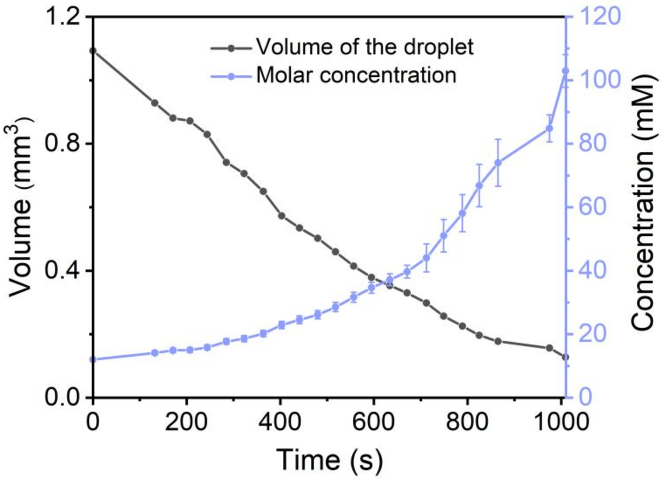

Figure S14. Temporal change of the volume and concentration of the levitating droplet contaning POM-$2C_{12}$/P123 aqueous solutions with a mixing ratio 3:1.

SAXS patterns of POM-$2C_{12}$/$C_{12}EO_8$ mixtures

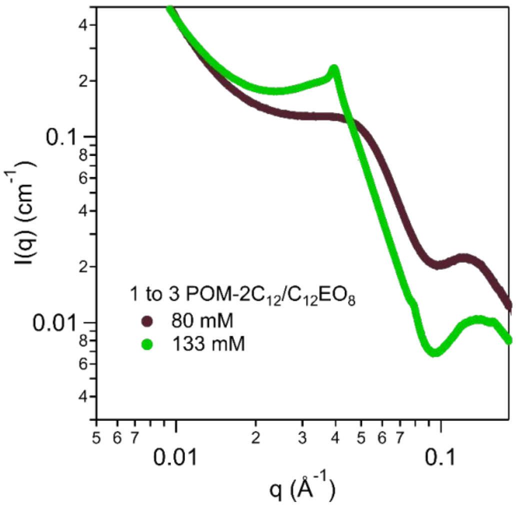

Figure S15. SAXS patterns of 80.0 and 133.0 mM of POM-$2C_{12}$/$C_{12}EO_8$ mixtures (mixing ratio 1:3).

Representative snapshots of DPD simulations results

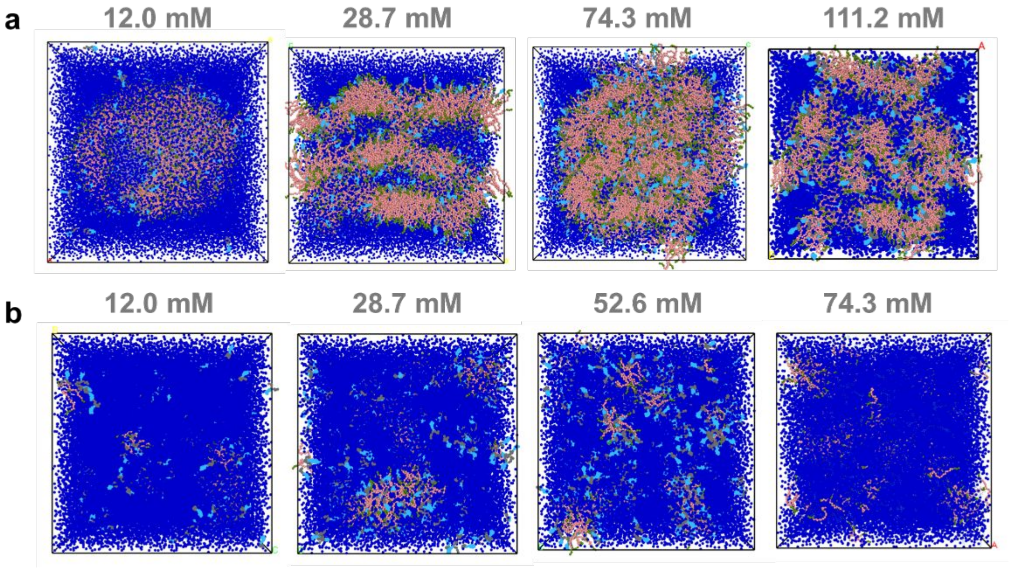

Figure S16. Representative snapshots from simulations on systems **a** POM-2C_{12}/P123 = 1:3, and **b** POM-2C_{12}/P123 = 3:1.

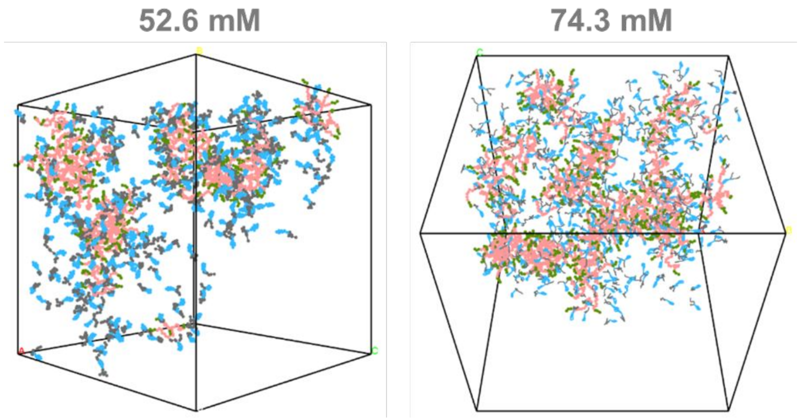

Figure S17. Snapshot of molecular dynamic simulations on POM-2C_{12}/P123 = 3:1 system with concentration at 52.6 and 74.3 mM. Some of the molecules are omitted for clarification. The POM units are coloured light blue, the PEO groups are coloured green and the PPO groups are coloured pink.

Dissipative Particle Dynamic Simulations

(1) DPD theory and parameters

Dissipative particle dynamics (DPD) method was firstly proposed by Hoogerbrugge and Koelman.[6,7] The method is based on the molecular dynamic simulation of coarse-grained particles called beads via soft interaction. In DPD method, a group of molecules or atoms is simplified as a bead, the size of the bead is larger than the atomic scale, but still small macroscopically. The beads dynamic evolution follows Newton's second law, and each bead bears four kinds of force as Equation 1.

$$F_i = \sum_{j \neq i} \left[f^C(r_{ij}) + f^D(r_{ij}, v_{ij}) + f^R(r_{ij}) + f^S(r_{ij}) \right] \qquad (1)$$

In which, $f^C(r_{ij})$, $f^D(r_{ij}, v_{ij})$, $f^R(r_{ij})$ are the conservative, dissipative, and random force respectively, $f^S(r_{ij})$ represent the spring force. The first three force belongs to nonbonding force, and the remaining one is bonding force. The solutions of the four forces are given by:

$$f^C(r_{ij}) = \left\{ \begin{array}{cc} a_{ij}\left(1 - \frac{r_{ij}}{r_C}\right)\hat{r_{ij}} & r_{ij} \le r_C \\ 0 & r_{ij} \ge r_C \end{array} \right\} \qquad (2)$$

$$f^D(r_{ij}, v_{ij}) = -\gamma \omega^D(r_{ij})\left(\hat{r_{ij}} \cdot v_{ij}\right)\hat{r_{ij}} \qquad (3)$$

$$f^R(r_{ij}) = \sigma \omega^R(r_{ij}) \xi_{ij} \Delta t^{-1/2} \hat{r_{ij}} \qquad (4)$$

$$f^S(r_{ij}) = -\, k_S(r_{ij} - r_0)\hat{r_{ij}} \qquad (5)$$

a_{ij} is called as conservative force parameter, the key parameter in DPD simulation, which indicates the interaction strength between particles i and j. r_{ij} and v_{ij} represent the distance and velocity between beads i and j. $\hat{r_{ij}}$ is a unit vector showing the direction of

r_{ij}. r_C is the cutoff radius which determines the interaction length in DPD simulation, γ and σ are the dissipative and random force parameters, respectively. The ξ_{ij} is the random number with a zero-mean distribution, and Δt is the time step. k_S is the spring constant, and r_0 is the equilibrium distance, which are used to describe the bonding force. The weight functions $\omega^D(r_{ij})$, $\omega^R(r_{ij})$, and σ, γ follows the relation:

$$\omega^D = (\omega^R)^2 \quad (6)$$

$$\sigma^2 = 2\gamma k_B T \quad (7)$$

Where the k_B is Boltzmann's constant, and T stands for the temperature. In the equations, the γ is commonly set as 4.5[8] which has been applied to get reasonable simulation results.

In order to determine the bead-bead interaction parameters, a_{ij}, Groot and Warren[9] introduced the Flory−Huggins interaction parameter χ_{ij}. The empirical correlation is the following formula:

$$a_{ij} = 25 + 3.50\chi_{ij} \quad (8)$$

Therefore, the key to describing interaction among beads is to obtain solubility parameter. Up to now, there are two methods of getting the χ_{ij} from theoretical calculations. One is obtained directly by Monte Carlo (MC), which can be achieved in Blends module of Material Studio. And another is indirectly calculated as eq 9:

$$\chi = \frac{V}{k_B T}(\delta_A - \delta_B)^2 \quad (9)$$

In which the V is bead volume, and the δ_i is the the solubility parameter of bead, which can be obtained through molecular dynamic simulation or experiment.

(2) Coarse-Grained method

The bead-bead interaction parameters in our simulation system are given in Table S1 based on eq (8)(9) and values provided in previous publications.[10,11]

The parameters for bond lengths and bending angles are listed in Table S2, which are obtained from explicit solvent (AES) MD simulations by inverting the Boltzmann method,[12] giving rise to comparable predictions with experimental results.

Table S4. Conservative force parameters among all beads

	C	E	P	W	water
C	25				
E	66.96	25			
P	25.00	66.96	25		
W	61.73	12.40	61.73	25	
water	61.73	26.05	61.73	12.40	25

Table S5. Intramolecular Parameters for Bonds and Bends.

Bond type	K_{bond}(kcal/mol/Å^2)	R0(Å)
E-E	30	3.2
E-P	20	3.52
P-P	25	3.36
Bend type	K_{bond}(kcal/mol/Å^2)	θ_0(deg)
E-E-E	10	128
E-E-P	7.5	119.25
E-P-P	7.5	119.25
P-P-P	5	110.5

(3) Simulation Details

DPD simulations were carried out by Material Studio software, and the periodic boundary conditions was applied in xyz directions. In this work, a total of 250,000 DPD simulation steps were carried out with a time step Δt =0.004τ. τ is DPD time scale, which corresponded to 14.657 ps. Therefore, the real simulation times is 250, 000 × 0.05863 = 14657.49 ps, which is enough to reach equilibrium. Moreover, the length scale, mass scale and energy scale of DPD simulation are 12.16 Å, 360 amu and 0.59219 kcal/mol, respectively.

Reference

(1) Di, A.; Schmitt, J.; da Silva, M. A.; Hossain, K. M. Z.; Mahmoudi, N.; Errington, R. J.; Edler, K. J. Self-Assembly of Amphiphilic Polyoxometalates for the Preparation of Mesoporous Polyoxometalate-Titania Catalysts. *Nanoscale* **2020**, *12* (43), 22245–22257.

(2) Droege, M. W. Ph.D Dissertation, University of Oregon, 1984.

(3) McDermott, A. G.; Budd, P. M.; McKeown, N. B.; Colina, C. M.; Runt, J. Physical Aging of Polymers of Intrinsic Microporosity: A SAXS/WAXS Study. *J. Mater. Chem. A* **2014**, *2* (30), 11742–11752.

(4) Berghausen, J.; Zipfel, J.; Lindner, P.; Richtering, W. Influence of Water-Soluble Polymers on the Shear-Induced Structure Formation in Lyotropic Lamellar Phases. *J. Phys. Chem. B* **2001**, *105* (45), 11081–11088.

(5) Feigin, L. A.; Svergun, D. I. *Structure Analysis by Small-Angle X-Ray and Neutron Scattering*; Plenum Press: New York, 1987.

(6) Koelman, J.; Hoogerbrugge, P. J. Dynamic Simulations of Hard-Sphere Suspensions under Steady Shear. *EPL (Europhysics Lett.* **1993**, *21* (3), 363.

(7) Hoogerbrugge, P. J.; Koelman, J. Simulating Microscopic Hydrodynamic Phenomena with Dissipative Particle Dynamics. *EPL (Europhysics Lett.* **1992**, *19* (3), 155.

(8) Ruiz-Morales, Y.; Mullins, O. C. Coarse-Grained Molecular Simulations to Investigate Asphaltenes at the Oil–Water Interface. *Energy & Fuels* **2015**, *29* (3), 1597–1609.

(9) Groot, R. D.; Warren, P. B. Dissipative Particle Dynamics: Bridging the Gap

between Atomistic and Mesoscopic Simulation. *J. Chem. Phys.* **1997**, *107* (11), 4423–4435.

(10) Liu, H.; Li, Y.; Krause, W. E.; Pasquinelli, M. A.; Rojas, O. J. Mesoscopic Simulations of the Phase Behavior of Aqueous EO19PO29EO19 Solutions Confined and Sheared by Hydrophobic and Hydrophilic Surfaces. *ACS Appl. Mater. Interfaces* **2012**, *4* (1), 87–95.

(11) Zhao, Y.; You, L.-Y.; Lu, Z.-Y.; Sun, C.-C. Dissipative Particle Dynamics Study on the Multicompartment Micelles Self-Assembled from the Mixture of Diblock Copolymer Poly (Ethyl Ethylene)-Block-Poly (Ethylene Oxide) and Homopolymer Poly (Propylene Oxide) in Aqueous Solution. *Polymer (Guildf).* **2009**, *50* (22), 5333–5340.

(12) Bedrov, D.; Ayyagari, C.; Smith, G. D. Multiscale Modeling of Poly (Ethylene Oxide)− Poly (Propylene Oxide)− Poly (Ethylene Oxide) Triblock Copolymer Micelles in Aqueous Solution. *J. Chem. Theory Comput.* **2006**, *2* (3), 598–606.
